# 4-Amino­phenyl­sulfur penta­fluoride

**DOI:** 10.1107/S160053680800024X

**Published:** 2008-01-09

**Authors:** Eva Lina Nava, Adolf Jesih, Evgeny Goreshnik

**Affiliations:** a Department of Inorganic Chemistry and Technology, Jozef Stefan Institute, Jamova 39 1000 Ljubljana, Slovenia

## Abstract

In the title compound, C_6_H_6_F_5_NS, the environment of the S atom is roughly octa­hedral. The axial F—S bond appears slightly elongated with respect to the four equatorial F—S bonds. Equatorial F atoms are staggered with respect to the benzene ring. The N atom is displaced from the benzene plane by 0.154 (4) Å. The F—S—C—C torsion angles differ greatly from the values observed in the related structure of 4-acetamido­phenyl­sulfur penta­fluoride. The packing is stabil­ized by weak N—H⋯F contacts.

## Related literature

For related literature, see: Raasch (1963 ▶); Bowden *et al.* (2000 ▶); Sheppard (1960 ▶, 1962 ▶).
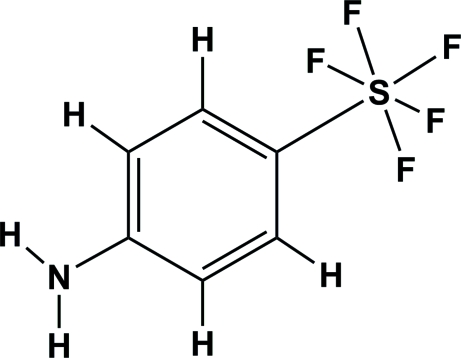


## Experimental

### 

#### Crystal data

C_6_H_6_F_5_NS
*M* *_r_* = 219.18
Orthorhombic, 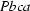

*a* = 16.0369 (13) Å
*b* = 5.7514 (5) Å
*c* = 17.5305 (15) Å
*V* = 1616.9 (2) Å^3^
*Z* = 8
Mo *K*α radiation
μ = 0.44 mm^−1^
*T* = 200 K
0.1 × 0.08 × 0.05 mm

#### Data collection

Rigaku Mercury CCDdiffractometer
Absorption correction: multi-scan (Blessing, 1995 ▶) *T*_min_ = 0.959, *T*_max_ = 0.981
6533 measured reflections
1650 independent reflections
633 reflections with *I* > 2σ(*I*)
*R*_int_ = 0.051

#### Refinement

*R*[*F* ^2^ > 2σ(*F* ^2^)] = 0.031
*wR*(*F* ^2^) = 0.065
*S* = 0.58
1650 reflections
118 parameters
H-atom parameters constrained
Δρ_max_ = 0.26 e Å^−3^
Δρ_min_ = −0.22 e Å^−3^

### 

Data collection: *CrystalClear* (Rigaku, 1999 ▶); cell refinement: *CrystalClear*; data reduction: *CrystalClear*; program(s) used to solve structure: *SIR92* (Altomare *et al.*, 1993 ▶); program(s) used to refine structure: *SHELXL97* (Sheldrick, 2008 ▶); molecular graphics: *ORTEPIII* (Burnett & Johnson, 1996 ▶) and *ORTEP-3 for Windows* (Farrugia, 1997 ▶); software used to prepare material for publication: *WinGX* (Farrugia, 1999 ▶) and *enCIFer* (Allen *et al.*, 2004 ▶).

## Supplementary Material

Crystal structure: contains datablocks global, I. DOI: 10.1107/S160053680800024X/dn2303sup1.cif

Structure factors: contains datablocks I. DOI: 10.1107/S160053680800024X/dn2303Isup2.hkl

Additional supplementary materials:  crystallographic information; 3D view; checkCIF report

## Figures and Tables

**Table 1:** Hydrogen-bond geometry (Å, °)

*D*—H⋯*A*	*D*—H	H⋯*A*	*D*⋯*A*	*D*—H⋯*A*
N1—H12⋯F5^i^	0.89	2.59	3.38	148

Symmetry code: (i) 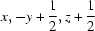.

**Fig. 1.**
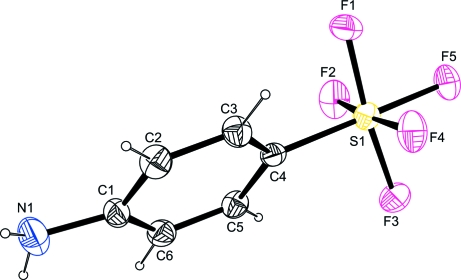
Molecular view of I with the atom-labelling scheme. Displacement ellipsoids are drawn at the 30% probability level. H atoms are represented as small sphers of arbitrary radii.

## supplementary crystallographic information

### Comment

Phenylsulfur pentafluorides were first synthesized (Sheppard, 1960) by the fluorination of aromatic disulfides with silver difluoride. Some SF_5_-benzene derivatives were patented as plant regulants, herbicides and bactericides (Raasch, 1963).

In the title compound, the environment of sulfur atom appears to be approximately octahedral (Fig. 1) with the C – S bond being 1.786 (3) Å, four equatorial S - F bonds of 1.577 (2) – 1.586 (2) Å and noticeably elongated to 1.600 (2) Å axial S – F bond. Equatorial F atoms are declined slightly away from the benzene ring resulting in the medium value of Feq – S – Fax angle of 86.9 °. Similar staggered conformation was observed earlier in the structure of 4-acetamidophenylsulfur pentafluoride (Bowden *et al.*, 2000). The F – S – C – C dihedral angles values of 43 and 47 ° differ from observed in above mentioned structure of 4-acetamidophenylsulfur pentafluoride 30 and 60 ° respectively. The packing is stabilized by weak N—H···F contacts.

### Experimental

Sample of 4-aminohenylsulfur pentafluoride was prepared in three steps according to original procedure (Sheppard, 1962). Bis-(4-nitrophenyl)-disulfide was fluorinated with silver difluoride in CFC113 solvent and the product 4-nitrophenylsulfur-pentafluoride was obtained in 10.0% yield and was consequently purified by preparative HPLC. 95% pure 4-nitrophenylsulfur pentafluoride was hydrogenated with hydrogen gas in acidic (HCL) ethanol solution, PtO_2_ was used as a catalyst. The 4-aminophenylsulfur pentafluoride hydrochloride obtained was reacted with sodium bicarbonate water solution and the product 4-aminophenylsulfur pentafluoride was extracted with diethyl ether and recrystallized from pentane. 4-Aminophenylsulfur pentafluoride crystallizes as white needles.

### Refinement

All H atoms attached to C atoms were fixed geometrically and treated as riding with C—H = 0.93 Å (aromatic) with *U*_iso_(H) = 1.2*U*_eq_(C). H atoms of amino group were located in difference Fourier maps and included in the subsequent refinement using restraints (N—H= 0.89 (1)Å and H···H= 1.57 (2) Å) with *U*_iso_(H) = 1.2*U*_eq_(N). In the last stage of refinement, they were treated as riding on their parent N atom.

### Crystal data

**Table:**

C_6_H_6_F_5_NS	*F*_000_ = 880
*M**_r_* = 219.18	*D*_x_ = 1.801 Mg m^−^^3^
Orthorhombic, *P**b**c**a*	Mo *K*α radiation λ = 0.71069 Å
Hall symbol: -P 2ac 2ab	Cell parameters from 71 reflections
*a* = 16.0369 (13) Å	θ = 1.2–29.1º
*b* = 5.7514 (5) Å	µ = 0.44 mm^−^^1^
*c* = 17.5305 (15) Å	*T* = 200 K
*V* = 1616.9 (2) Å^3^	Chunk, colourless
*Z* = 8	0.1 × 0.08 × 0.05 mm

### Data collection

**Table:**

Mercury CCD (2x2 bin mode) diffractometer	*R*_int_ = 0.051
dtprofit.ref scans	θ_max_ = 26.4º
Absorption correction: multi-scan(Blessing, 1995)	θ_min_ = 2.3º
*T*_min_ = 0.959, *T*_max_ = 0.981	*h* = 0→20
6533 measured reflections	*k* = 0→7
1650 independent reflections	*l* = 0→21
633 reflections with *I* > 2σ(*I*)	

### Refinement

**Table:**

Refinement on *F*^2^	Secondary atom site location: difference Fourier map
Least-squares matrix: full	Hydrogen site location: inferred from neighbouring sites
*R*[*F*^2^ > 2σ(*F*^2^)] = 0.031	H-atom parameters constrained
*wR*(*F*^2^) = 0.065	*w* = 1/[σ^2^(*F*_o_^2^)]
*S* = 0.58	(Δ/σ)_max_ = 0.001
1650 reflections	Δρ_max_ = 0.26 e Å^−^^3^
118 parameters	Δρ_min_ = −0.22 e Å^−^^3^
Primary atom site location: structure-invariant direct methods	Extinction correction: none

### Special details

**Table:**

Geometry. All e.s.d.'s (except the e.s.d. in the dihedral angle between two l.s. planes) are estimated using the full covariance matrix. The cell e.s.d.'s are taken into account individually in the estimation of e.s.d.'s in distances, angles and torsion angles; correlations between e.s.d.'s in cell parameters are only used when they are defined by crystal symmetry. An approximate (isotropic) treatment of cell e.s.d.'s is used for estimating e.s.d.'s involving l.s. planes.

### Fractional atomic coordinates and isotropic or equivalent isotropic displacement parameters (Å^2^)

**Table:**

	*x*	*y*	*z*	*U*_iso_*/*U*_eq_	
S1	0.89753 (5)	0.08028 (15)	0.09772 (6)	0.0401 (2)	
F1	0.87251 (10)	0.3449 (3)	0.10747 (12)	0.0606 (6)	
F2	0.80397 (8)	0.0204 (3)	0.07597 (11)	0.0597 (6)	
F3	0.92307 (9)	−0.1787 (3)	0.07811 (11)	0.0529 (6)	
F4	0.99217 (9)	0.1448 (3)	0.10997 (12)	0.0566 (6)	
F5	0.91119 (10)	0.1370 (3)	0.00922 (11)	0.0608 (6)	
C1	0.84866 (18)	−0.0742 (6)	0.3495 (2)	0.0417 (9)	
C2	0.89380 (16)	0.1215 (6)	0.3276 (2)	0.0424 (9)	
H2	0.9136	0.2228	0.3648	0.051*	
C3	0.90968 (17)	0.1681 (5)	0.2514 (2)	0.0391 (9)	
H3	0.9394	0.3005	0.2377	0.047*	
C4	0.88150 (16)	0.0181 (5)	0.19639 (18)	0.0303 (8)	
C5	0.83895 (16)	−0.1814 (5)	0.2167 (2)	0.0364 (8)	
H5	0.8205	−0.2842	0.1794	0.044*	
C6	0.82413 (17)	−0.2267 (5)	0.2925 (2)	0.0415 (9)	
H6	0.7969	−0.3635	0.3059	0.050*	
N1	0.82569 (15)	−0.1078 (5)	0.42463 (17)	0.0618 (9)	
H11	0.8081	−0.2512	0.4320	0.074*	
H12	0.8522	−0.0289	0.4603	0.074*	

### Atomic displacement parameters (Å^2^)

**Table:**

	*U*^11^	*U*^22^	*U*^33^	*U*^12^	*U*^13^	*U*^23^
S1	0.0450 (5)	0.0386 (5)	0.0367 (6)	0.0025 (4)	0.0026 (5)	−0.0008 (5)
F1	0.0980 (14)	0.0353 (11)	0.0486 (15)	0.0151 (10)	0.0129 (12)	0.0070 (11)
F2	0.0412 (10)	0.0926 (15)	0.0453 (15)	−0.0039 (9)	−0.0090 (10)	−0.0005 (12)
F3	0.0731 (12)	0.0369 (11)	0.0487 (15)	0.0077 (9)	0.0085 (11)	−0.0127 (10)
F4	0.0432 (10)	0.0714 (13)	0.0552 (16)	−0.0147 (9)	0.0108 (10)	−0.0010 (12)
F5	0.0819 (13)	0.0706 (14)	0.0300 (13)	0.0071 (10)	0.0147 (11)	0.0067 (11)
C1	0.040 (2)	0.053 (2)	0.032 (2)	0.0120 (17)	0.0036 (18)	0.009 (2)
C2	0.0417 (19)	0.047 (2)	0.038 (2)	−0.0027 (17)	−0.0073 (18)	−0.0070 (19)
C3	0.0419 (19)	0.037 (2)	0.038 (2)	−0.0076 (15)	0.0024 (18)	−0.0009 (19)
C4	0.0336 (17)	0.0276 (18)	0.030 (2)	0.0041 (14)	0.0002 (15)	−0.0005 (16)
C5	0.0362 (18)	0.0312 (19)	0.042 (2)	−0.0041 (15)	0.0018 (17)	−0.0071 (19)
C6	0.0424 (19)	0.033 (2)	0.049 (3)	−0.0003 (16)	0.0081 (19)	0.004 (2)
N1	0.0770 (19)	0.068 (2)	0.040 (2)	−0.0009 (16)	0.0052 (17)	0.0081 (19)

### Geometric parameters (Å, °)

**Table:**

S1—F4	1.5771 (16)	C2—H2	0.9300
S1—F3	1.5826 (17)	C3—C4	1.370 (4)
S1—F1	1.5832 (17)	C3—H3	0.9300
S1—F2	1.5860 (16)	C4—C5	1.382 (4)
S1—F5	1.600 (2)	C5—C6	1.375 (4)
S1—C4	1.785 (3)	C5—H5	0.9300
C1—N1	1.381 (4)	C6—H6	0.9300
C1—C6	1.386 (4)	N1—H11	0.8813
C1—C2	1.392 (4)	N1—H12	0.8823
C2—C3	1.386 (4)		
			
F4—S1—F3	90.11 (9)	C3—C2—C1	121.2 (3)
F4—S1—F1	90.18 (10)	C3—C2—H2	119.4
F3—S1—F1	173.62 (13)	C1—C2—H2	119.4
F4—S1—F2	173.86 (13)	C4—C3—C2	119.7 (3)
F3—S1—F2	89.33 (10)	C4—C3—H3	120.1
F1—S1—F2	89.70 (10)	C2—C3—H3	120.1
F4—S1—F5	87.26 (10)	C3—C4—C5	120.2 (3)
F3—S1—F5	86.90 (11)	C3—C4—S1	120.6 (2)
F1—S1—F5	86.75 (11)	C5—C4—S1	119.2 (3)
F2—S1—F5	86.60 (11)	C6—C5—C4	119.5 (3)
F4—S1—C4	93.09 (12)	C6—C5—H5	120.2
F3—S1—C4	93.40 (12)	C4—C5—H5	120.2
F1—S1—C4	92.95 (13)	C5—C6—C1	121.8 (3)
F2—S1—C4	93.05 (12)	C5—C6—H6	119.1
F5—S1—C4	179.54 (13)	C1—C6—H6	119.1
N1—C1—C6	121.5 (3)	C1—N1—H11	110.9
N1—C1—C2	121.0 (4)	C1—N1—H12	118.5
C6—C1—C2	117.4 (3)	H11—N1—H12	122.1

### Hydrogen-bond geometry (Å, °)

**Table:**

*D*—H···*A*	*D*—H	H···*A*	*D*···*A*	*D*—H···*A*
N1—H12···F5^i^	0.89	2.59	3.38	148

Symmetry codes: (i) *x*, −*y*+1/2, *z*+1/2.
